# Antibacterial potential of *Stenotrophomonas maltophilia* complex cystic fibrosis isolates

**DOI:** 10.1128/msphere.00335-24

**Published:** 2024-07-09

**Authors:** Cristian V. Crisan, Morgan L. Pettis, Joanna B. Goldberg

**Affiliations:** 1 Department of Pediatrics, Division of Pulmonary, Asthma, Cystic Fibrosis, and Sleep, Emory University School of Medicine, Atlanta, Georgia, USA; 2 Emory+Children’s Center for Cystic Fibrosis and Airway Disease Research, Emory University School of Medicine, Atlanta, Georgia, USA; 3 Department of Biology, Emory University, Atlanta, Georgia, USA; Hackensack Meridian Health Center for Discovery and Innovation, Nutley, New Jersey, USA

**Keywords:** *Stenotrophomonas maltophilia*, polymicrobial interactions, antibacterial, emerging pathogen, cystic fibrosis

## Abstract

Over 160,000 people worldwide suffer from cystic fibrosis (CF), a genetic condition that causes mucus to accumulate in internal organs. Lung decline is a significant health burden for people with CF (pwCF), and chronic bacterial pulmonary infections are a major cause of death. *Stenotrophomonas maltophilia* complex (Smc) is an emerging, multidrug-resistant CF pathogen that can cause pulmonary exacerbations and result in higher mortality. However, little is known about the antagonistic interactions that occur between Smc isolates from pwCF and competitor bacteria. We obtained 13 Smc isolates from adult and pediatric pwCF located in the United States or Australia. We co-cultured these isolates with *Pseudomonas aeruginosa*, *Staphylococcus aureus,* and *Escherichia coli*. We also performed whole-genome sequencing of these Smc isolates and compared their genomes using average nucleotide identity analyses. We observed that some Smc CF isolates can engage in antagonistic interactions with *P. aeruginosa* and *S. aureus* but recovered a substantial number of *P. aeruginosa* and *S. aureus* cells following co-cultures with all tested Smc isolates. By contrast, we discovered that most Smc CF isolates display strong antibacterial properties against *E. coli* cells and reduce recovery below detectable limits. Finally, we demonstrate that Smc CF strains from this study belong to diverse phylogenetic lineages.

**IMPORTANCE:**

Antagonism toward competitor bacteria may be important for the survival of *Stenotrophomonas maltophilia* complex (Smc) in external environments, for the elimination of commensal species and colonization of upper respiratory tracts to enable early infections, and for competition against other pathogens after establishing chronic infections. These intermicrobial interactions could facilitate the acquisition of Smc by people with cystic fibrosis from environmental or nosocomial sources. Elucidating the mechanisms used by Smc to eliminate other bacteria could lead to new insights into the development of novel treatments.

## OBSERVATION

Cystic fibrosis (CF) is a genetic condition that affects approximately 160,000 people worldwide (1). People with CF (pwCF) suffer from mucus accumulation in their organs. In the respiratory tract, this creates a favorable environment for opportunistic bacterial pathogens to establish chronic pulmonary infections that lead to lung function decline (2). *Pseudomonas aeruginosa* and *Staphylococcus aureus* are the most common CF pathogens; *P. aeruginosa* is found in ~30%–60% of adult pwCF, while *S. aureus* is isolated from ~50% to 75% of pediatric pwCF (3). Relatively little is known about the roles of *Escherichia coli* in CF, but the prevalence of the bacterium in the respiratory tracts of people with pwCF has been reported to be ~12%–34% (4–7). *E. coli* isolates from pwCF can cause persistent pulmonary infections, can display phenotypes associated with virulence such as capsule formation and hemolysis, and belong to phylogenetic groups that contain virulent strains (6, 7).

*Stenotrophomonas maltophilia* is a globally distributed, emerging, and multidrug-resistant gram-negative bacterial pathogen that infects ~5%–30% of pwCF (8, 9). pwCF colonized with this bacterium may have a threefold higher mortality rate, a higher risk of requiring antibiotics, and more frequent pulmonary exacerbations (10, 11). The CF Foundation Infection Research Initiative recently identified *S. maltophilia* as an “area of need” for research (12). *S. maltophilia* also infects immunocompromised, cancer, COVID-19, and chronic obstructive pulmonary disease patients (8, 13, 14). Bloodstream infections can have mortality rates above 65% (15). The bacterium is found in natural environments, urban locations, and is a common source of nosocomial infections (8, 16).

*S. maltophilia* exhibits a high degree of phylogenetic complexity and has been classified into 23 distinct lineages (17). Most sequenced *S. maltophilia* isolates belong to the Sm6 “*sensu stricto*” lineage; other “Sm” lineages are designated as “*sensu lato,*” while Sgn lineages are the most divergent from *sensu stricto* (17). The term *“S. maltophilia* complex” (Smc) is used for isolates that belong to any of the 23 lineages (17). Strains within each lineage share >95% average nucleotide identity (ANI) (17).

During infections and in environmental settings, Smc competes with other bacteria. Antagonistic microbial interactions that occur during polymicrobial infections can affect expression of virulence factors, antibiotic resistance, immune system evasion, and disease outcomes (18, 19). pwCF that harbor Smc are often co-infected with *P. aeruginosa* and/or *S. aureus*, but little is known about antagonistic interactions that occur between these CF bacterial pathogens (20, 21). Previous studies have reported that Smc strains can engage in both antagonistic and cooperative interactions with *P. aeruginosa* (22–27).

Secreted metabolites and contact-dependent protein secretion systems are used by bacteria to eliminate competitors (28). The type IV secretion system (T4SS) and type VI secretion system (T6SS) are distinct antibacterial weapons that deliver toxic proteins in a contact-dependent manner into bacterial cells (28). *virB10* and *virD4* genes are essential for T4SS activity, while *tssC* and *tssM* genes are required for T6SS activity (25, 27, 28). Smc strain K279a possesses an antibacterial T4SS (25, 27), while Smc strain STEN00241 encodes an antibacterial T6SS (24). However, neither of these strains were obtained from pwCF. We hypothesized that Smc strains obtained from pwCF can also eliminate competitor bacteria and harbor T4SS or T6SS genes.

To evaluate the ability of Smc CF strains to antagonize other bacteria, we obtained 11 strains from the Emory University Cystic Fibrosis Biospecimen Registry (Atlanta, USA) and 2 strains from the Adult Cystic Fibrosis Centre at the Prince Charles Hospital (Brisbane, Queensland, Australia) (Table S1). Each Smc strain from this study was obtained from a different pwCF. Nine isolates were obtained from adult pwCF and four from pediatric pwCF (Table S1). Six Smc isolates were obtained from pwCF co-infected with *P. aeruginosa* at the time of collection, five from pwCF co-infected with *S. aureus*, and a single isolate from a pwCF co-infected with both *P. aeruginosa* and *S. aureus* (Table S1).

**Fig 1:**
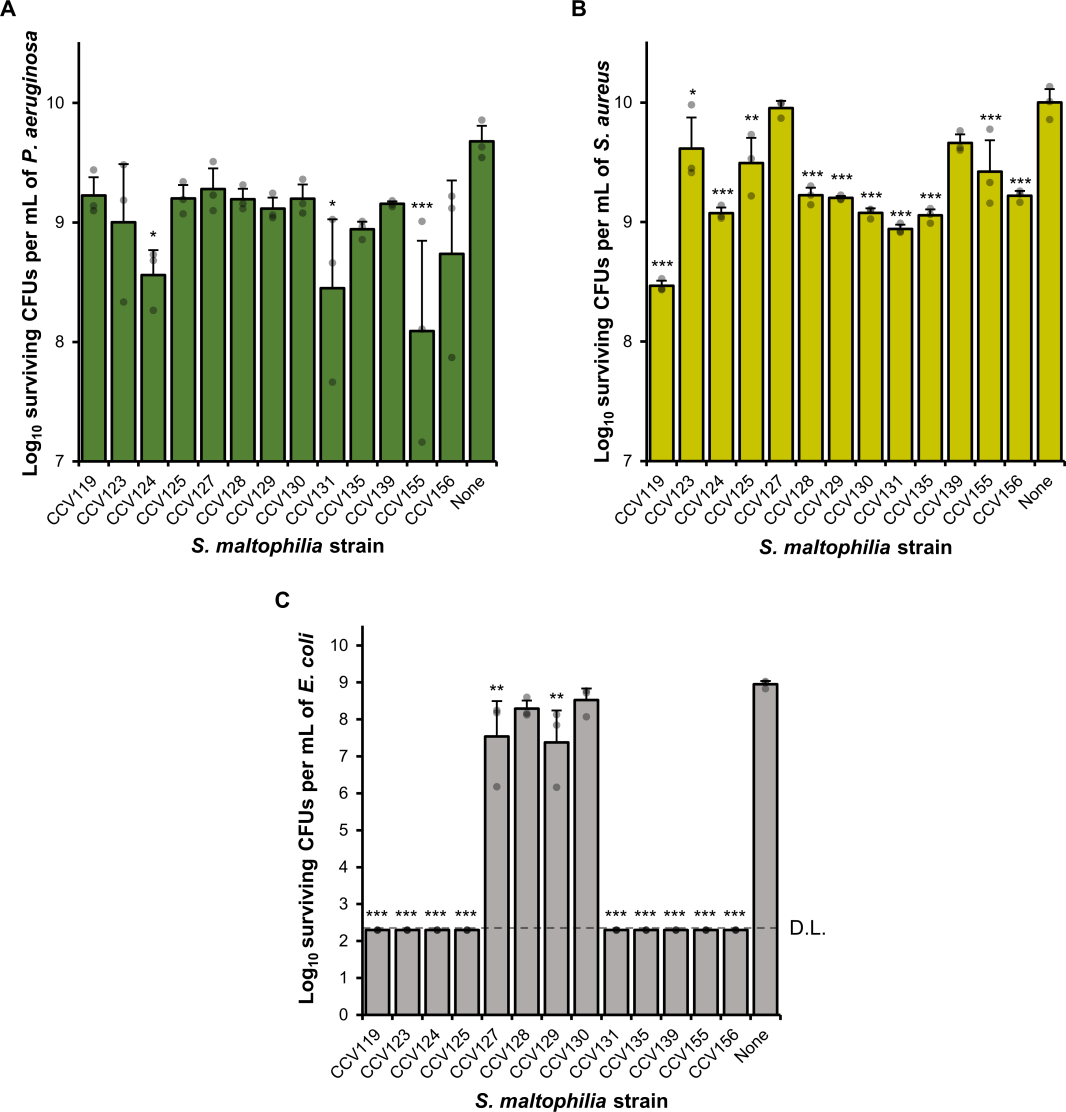
Survival of *P. aeruginosa* PAO1, *S. aureus* JE2, and *E. coli* DH5α following co-cultures with Smc CF isolates. The indicated Smc strains were co-cultured on solid LB medium with competitor *P. aeruginosa* PAO1 (**A**), *S. aureus* JE2 (**B**), or *E. coli* DH5α (**C**) at a ratio of 10:1 (Smc:competitor) and incubated at 37°C for 22 hours. The number of surviving *P. aeruginosa, S. aureus, and E. coli* colony-forming units (CFUs) per milliliter was determined. D.L., detection limit. A one-way ANOVA with Dunnett’s post hoc test was used to determine significance between the recovered CFUs of each co-culture and the recovered CFUs of the *P. aeruginosa, S. aureus,* or *E. coli* monocultures, respectively. ***P < 0.001, **P < 0.01, *P < 0.05. Unlabeled comparisons were not statistically significant. Three biological replicates were performed for each co-culture. Error bars represent standard deviations, solid bars represent averages, and dots represent biological replicates. Please see the Supplemental Material for detailed co-culture methods.

We co-cultured each Smc CF isolate with the common laboratory strains of *P. aeruginosa* or *S. aureus*, PAO1 or JE2, respectively. We observed that three isolates (CCV124, CCV131, and CCV155) significantly reduced recovery of *P. aeruginosa* by ~1–2 logs, but more than 10^8^ *P. aeruginosa* cells/mL were recovered on average following co-cultures with all Smc isolates (Fig. 1A). Even though most Smc strains significantly reduced *S. aureus* recovery, more than 10^8^ cells/mL *S*. *aureus* cells were recovered following co-cultures with each Smc CF isolate (Fig. 1B). These results suggest that Smc strains from pwCF may compete against but do not completely eradicate *P. aeruginosa* or *S. aureus*.

We discovered that most Smc strains (9/13) reduced the number of *E. coli* cells below the detection limit (Fig. 1C). Two isolates from adult pwCF (CCV127 and CCV129) had a lower impact on the number of recovered *E. coli* cells during co-cultures, while two other Smc isolates (CCV128 and CCV130, also from adult pwCF) did not significantly affect the number of recovered *E. coli* during co-cultures (Fig. 1C). In total, CCV124, CCV131, and CCV155 significantly reduced survival of *P. aeruginosa*, *S. aureus*, and *E. coli* (Table S1).

To examine if Smc CF strains can be inhibited by these other bacteria, we performed co-cultivation assays on agar medium. We detected limited inhibitory effects of *P. aeruginosa* against some Smc strains, but we did not observe any inhibitory effects for *E. coli* or *S. aureus* (Fig. S1).

**Fig 2:**
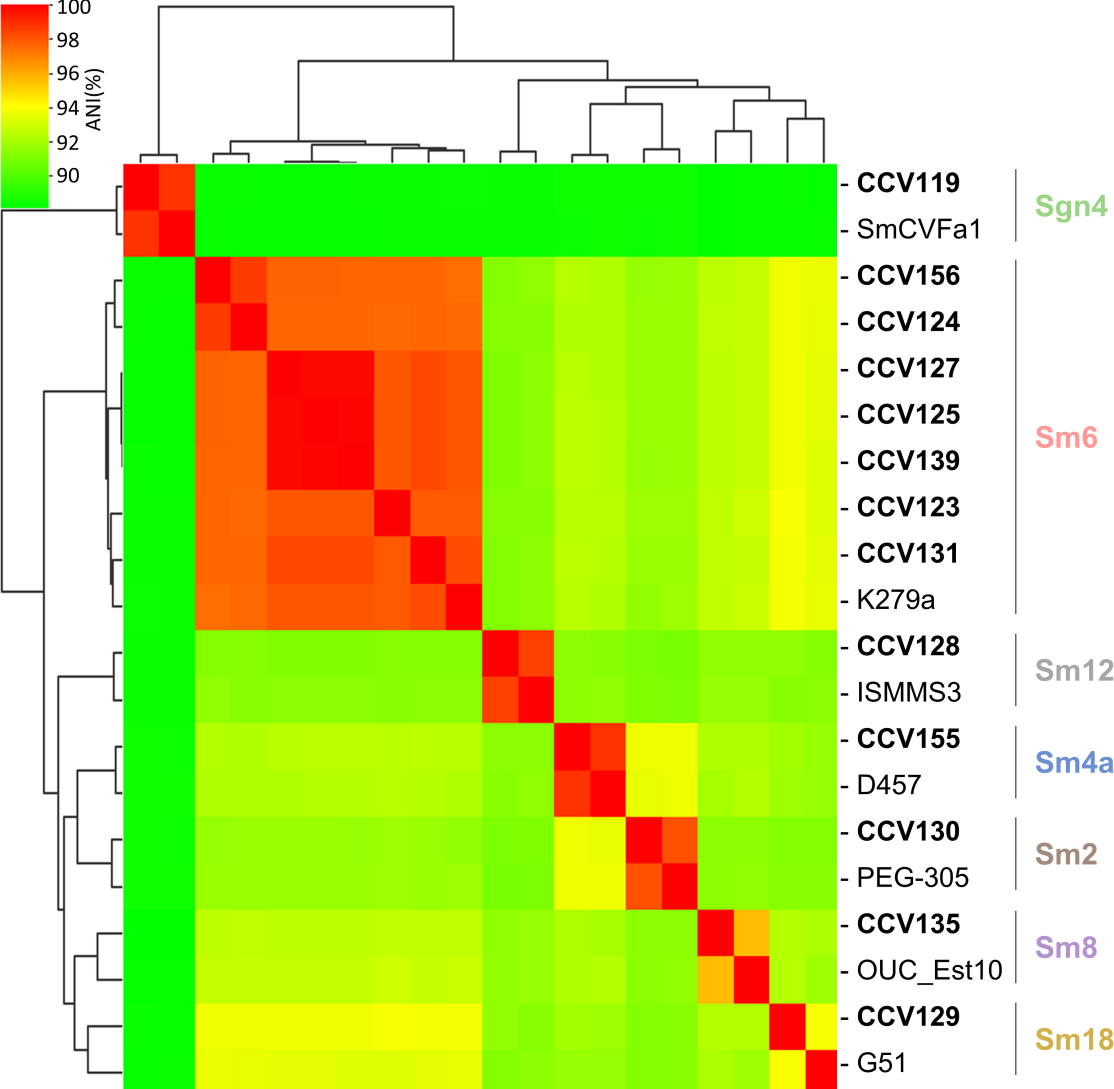
Phylogenetic analysis of *S. maltophilia* CF isolates tested here. Following Illumina whole-genome sequencing, an average nucleotide identity matrix was computed using ANIclustermap. Strain names sequenced here are shown in bold. Publicly available Smc genome sequences for strains PEG-305 (Sm2), D457 (Sm4a), K279a (Sm6), OUC_Est10 (Sm8), ISMMS3 (Sm12), G51 (Sm18), and SmCVFa1 (Sgn4) were included in the analysis as representatives of their respective lineages.

To analyze the phylogenetic diversity of the Smc strains from this study, we performed Illumina whole-genome sequencing. We observed that, similar to other Smc genomes analyzed (17), those that we sequenced have a size of approximately 4–5.2 million base pairs and a GC content of ~66%–67% (Table S2). Gröschel et al. found that ~32% of all publicly available Smc genomes are from the Sm6 lineage and that the Smc lineages Sm6, Sm2, and Sm13 are significantly associated with human respiratory tracts (17). In contrast, the more distantly related Sgn1, Sgn2, Sgn3, and Sm11 lineages are more likely to be isolated from environmental sources (17). Based on the ANI, seven isolates (~54%) from our study belong to the *sensu stricto* Sm6 lineage (Fig. 2; Fig. S2). Strains CCV125, CCV127, and CCV139 have an ANI >99.7%, even though they were isolated from different pwCF in different months. Five isolates belong to a *sensu lato* lineage, while a single isolate belongs to an Sgn lineage. Except for CCV129, all isolates from this study have >95% ANI with an Smc isolate from a known lineage (Fig. 2; Fig. S2). CCV129 shares 93%–94% ANI with G51 (from the Sm18 lineage), indicating that its classification into an established Smc lineage might be less clear.

Taken together, our results suggest that most Smc CF strains have the potential to reduce the growth of some heterologous bacterial cells. We did not observe a correlation between the co-infection status of Smc CF isolates and their ability to antagonize other bacteria. We hypothesize that antagonistic interactions help Smc strains survive during polymicrobial infections by competing with other CF pathogens. Our conclusions support findings which have shown that Smc is often co-isolated with *P. aeruginosa* and *S. aureus* and can grow in polymicrobial biofilms with these bacteria (20, 21, 29). *P. aeruginosa* employs multiple defense systems (including stress response pathways) against aggression from other bacteria as well as offensive contact-dependent secretion systems and diffusible metabolites that allow it to eliminate other bacteria (28, 30). These mechanisms might play important roles in protecting *P. aeruginosa* from Smc. Clinical studies have reported that antibiotic treatment in pwCF also resulted in a ~1–2 log *P. aeruginosa* density reduction (31, 32). This reduction may be correlated with improved lung function and decreased inflammation (31). The thick peptidoglycan layer may contribute to the defensive properties of *S. aureus* against Smc. Differences in growth rates of the Smc CF isolates tested here are also likely to affect their ability to impair the growth of competitor cells.

Two isolates from this study (CCV119 and CCV128) possess *tssC* and *tssM* T6SS genes but do not have *virB10* or *virD4* T4SS genes (Table S2). Except for CCV130, which has a *virD4* but not a *virB10* homolog, all other Smc strains examined possess *virB10* and *virD4* genes but not *tssC* or *tssM* genes (Table S2). We did not observe a clear association between the prevalence of T4SS or T6SS genes and an isolate’s antibacterial potential. It is possible that even if a strain encodes T4SS or T6SS components, these systems are not expressed in the tested conditions, or competitor species may resist the toxic proteins delivered by these systems.

The antibacterial properties of Smc may be important during initial airway colonization, perhaps to outcompete commensal species from the upper respiratory tract and facilitate early infections (28, 33). Furthermore, the ability of Smc to antagonize other bacteria could allow this pathogen to survive in external environments and could increase the probability that pwCF acquire an infection (28). It is unknown whether Smc strains from sources other than pwCF differ in their antibacterial potential compared to the isolates from this study.

Our genome analyses suggest that isolates from our study are representative of the overall phylogenetic distribution of Smc. These results are in accordance with recent studies, which have also reported that Smc CF isolates belong to multiple lineages (34). We did not observe a direct correlation between the phylogenetic lineages of Smc isolates and their antibacterial properties.

Our study has some limitations. First, co-cultures were performed on solid agar surfaces; experiments where co-cultures are performed in flow chambers (which may better mimic the physical conditions from the respiratory tracts of pwCF), on synthetic cystic fibrosis sputum medium, on cell lines, or in animal models could be employed to examine the potential roles played by external factors in affecting the survival of target bacteria (29). Second, the target strains of *P. aeruginosa*, *S. aureus,* and *E. coli* were standard laboratory strains. Additional experiments are required to determine if the Smc CF isolates tested here can also antagonize *P. aeruginosa*, *S. aureus*, and *E. coli* strains obtained from the respiratory tracts of pwCF. It is possible that different co-culture times between Smc and competitor bacteria and different Smc:competitor ratios could provide important insights into the spatio-temporal dynamics of the observed antagonistic interactions.

Future studies will examine the roles played by the T4SS and T6SS in conferring Smc CF isolates the ability to antagonize bacteria, the impact of regulatory factors such as quorum sensing on the antagonistic interactions between Smc and competitor cells, and the potential roles played by these systems in modulating virulence. Understanding the mechanisms behind the antibacterial properties of Smc isolates could lead to the design of novel therapeutics and treatment strategies.

## Data Availability

Genome sequences for strains from this study are available under the NCBI BioProject PRJNA1052082. The following accession numbers correspond to the *Stenotrophomonas maltophilia* complex genomes sequenced in this study: GCA_034491205.1 (CCV119), GCA_034492185.1 (CCV123), GCA_034492385.1 (CCV124), GCA_034491985.1 (CCV125), GCA_036419455.1 (CCV127), GCA_034491325.1 (CCV128), GCA_034491245.1 (CCV129), GCA_034506555.1 (CCV130), GCA_034491305.1 (CCV131), GCA_034491285.1 (CCV135), GCA_034491265.1 (CCV139), GCA_034506535.1 (CCV155), GCA_034491225.1 (CCV156)

## SUPPLEMENTAL MATERIAL

Supplemental Figures msphere.00335-24-s0001.docx Figures S1 and S2.

Supplemental Material msphere.00335-24-s0002.docx Supplemental text.

Supplemental Tables msphere.00335-24-s0003.docx Tables S1 and S2.

ASM does not own the copyrights to Supplemental Material that may be linked to, or accessed through, an article. The authors have granted ASM a non-exclusive, world-wide license to publish the Supplemental Material files. Please contact the corresponding author directly for reuse.
