# Supplementary material for: Antibacterial potential of Stenotrophomonas maltophilia complex cystic fibrosis isolates
Source: mSphere. 2024 Jul 9;9(7):e00335-24. doi: 10.1128/msphere.00335-24 (PMC11288042; doi:10.1128/msphere.00335-24)
Supplement: Supplemental Figures — Figures S1 and S2. [file msphere.00335-24-s0001.docx]

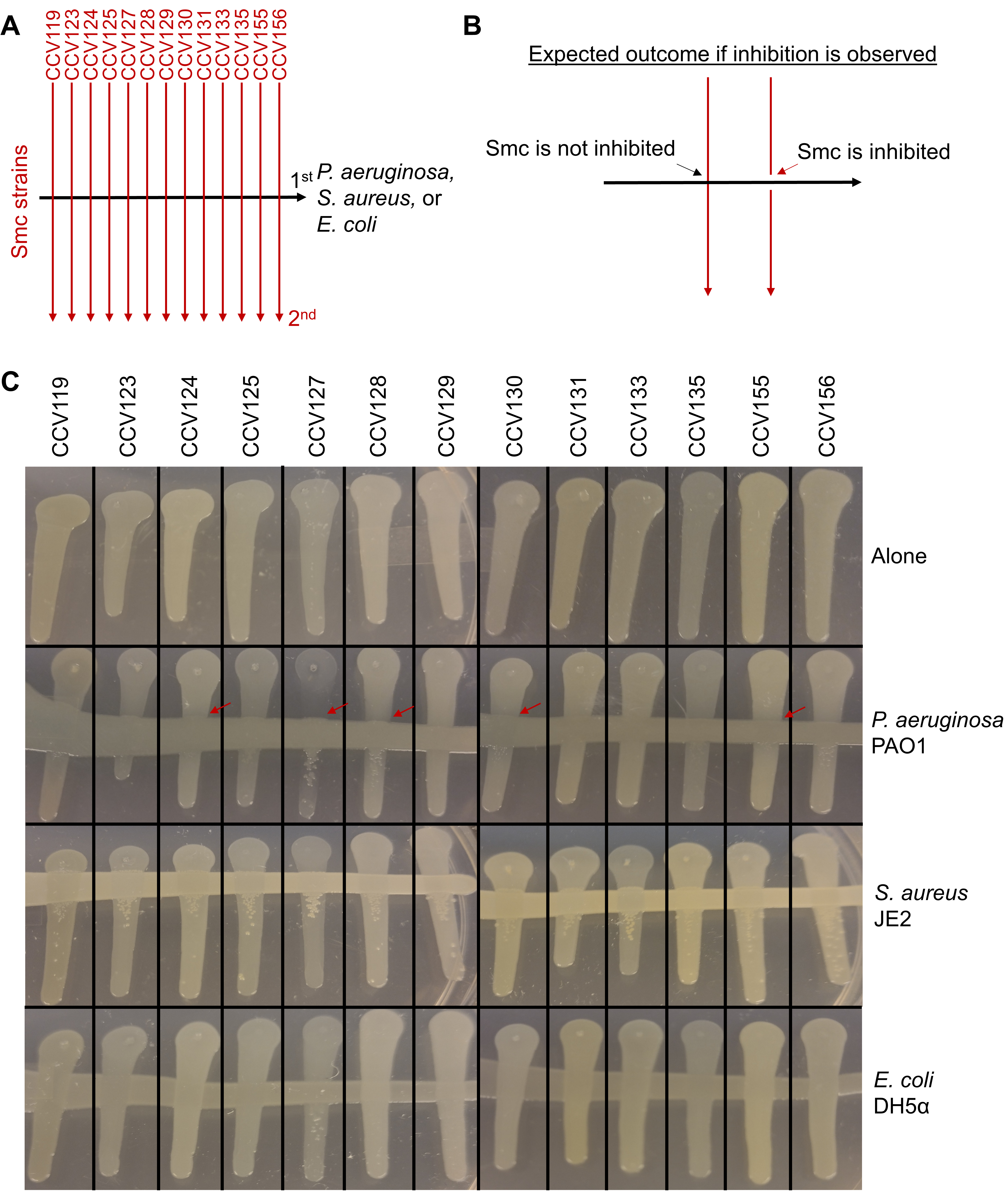


**Supplementary Figure 1. Inhibition co-culture assay between Smc strains and *P. aeruginosa* PAO1, *S. aureus* JE2, and *E. coli* DH5α.** First, *P. aeruginosa* PAO1, *S. aureus* JE2, or *E. coli* DH5α cells were set to an OD_600_ = 0.1 and spread (from left to right) on LB agar medium. Second, the indicated Smc strains were set to an OD_600_ = 0.1 and spread in a line perpendicular to *P. aeruginosa*, *S. aureus,* or *E. coli*. Zones of clearing indicative of Smc growth inhibition were examined at co-culture interfaces and are indicated by red arrows.

**
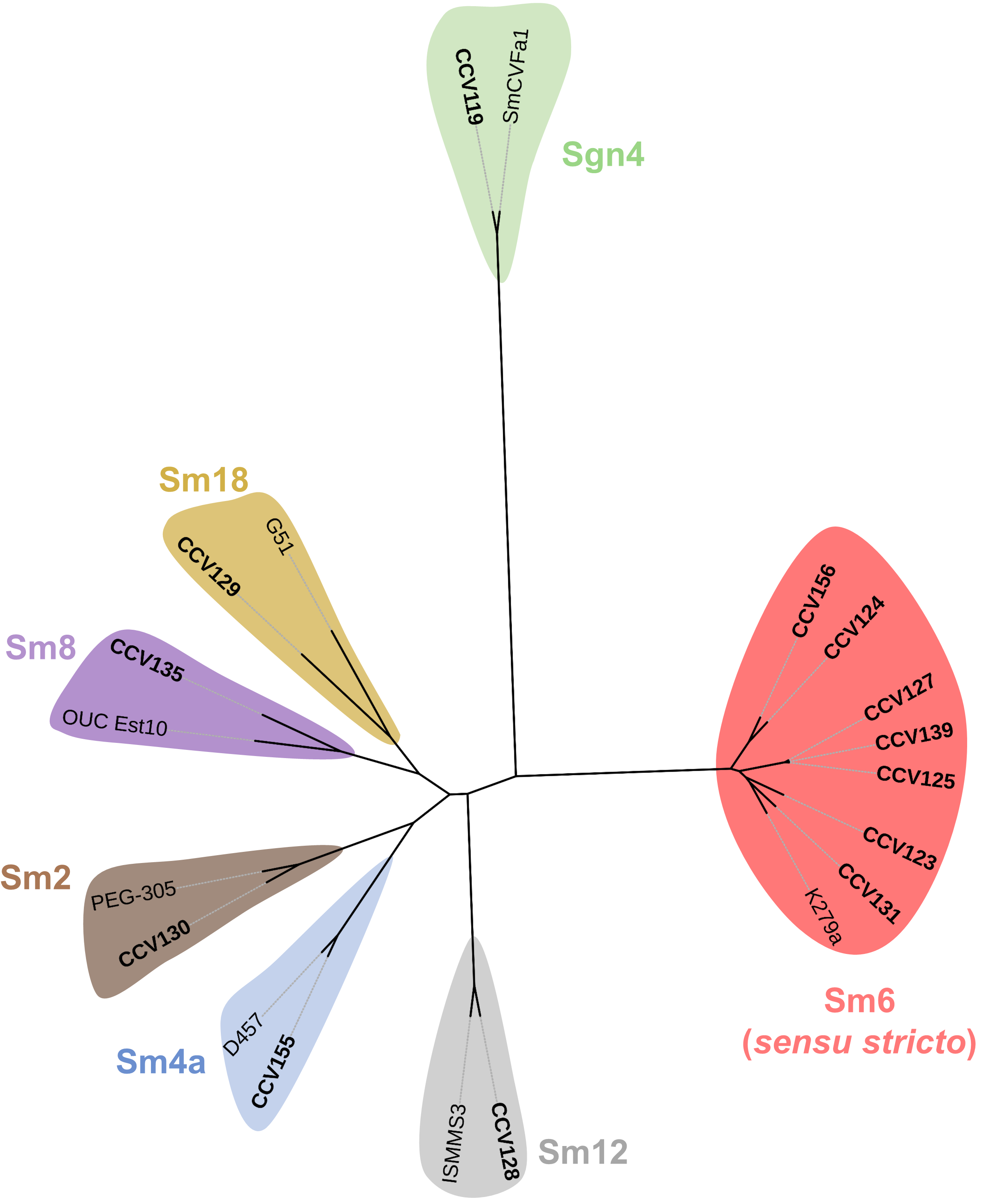
**

**Supplementary Figure 2. Phylogenetic tree of Smc genomes from this study and representative Smc strains from the indicated linages.** An ANI phylogenetic tree for the indicated Smc strains was built in iTol (1). Strain names sequenced here are shown in bold. Publicly available Smc genome sequences for strains PEG-305 (Sm2), D457 (Sm4a), K279a (Sm6), OUC_Est10 (Sm8), ISMMS3 (Sm12), G51 (Sm18), and SmCVFa1 (Sgn4) were included in the analysis as representatives of their respective lineages.

**Reference**

1. Letunic I, Bork P. 2021. Interactive Tree Of Life (iTOL) v5: an online tool for phylogenetic tree display and annotation. Nucleic Acids Res 49:W293–W296.
