# Supplementary material for: Antibacterial potential of Stenotrophomonas maltophilia complex cystic fibrosis isolates
Source: mSphere. 2024 Jul 9;9(7):e00335-24. doi: 10.1128/msphere.00335-24 (PMC11288042; doi:10.1128/msphere.00335-24)
Supplement: Supplemental Material — Supplemental text. [file msphere.00335-24-s0002.docx]

**Supplementary Material**

**Antibacterial potential of *Stenotrophomonas maltophilia* complex cystic fibrosis isolates**

Cristian V. Crisan^1,2^, Morgan Pettis^3^, and Joanna B. Goldberg^1,2,*^

^1^Department of Pediatrics, Division of Pulmonary, Asthma, Cystic Fibrosis, and Sleep, Emory University School of Medicine, Atlanta, Georgia, USA

^2^Emory+Children's Center for Cystic Fibrosis and Airway Disease Research, Emory University School of Medicine, Atlanta, Georgia, USA

^3^Department of Biology, Emory University, Atlanta, Georgia, USA

**Materials and Methods**

**Genomic DNA extraction and Illumina Whole Genome Sequencing**

*S. maltophilia* complex (Smc) isolates from the Emory University Cystic Fibrosis Biospecimen Registry (Atlanta, USA) and the Adult Cystic Fibrosis Centre at the Prince Charles Hospital (Brisbane, Queensland, Australia) (Table 1) were streaked on LB plates containing imipenem (20 µg/mL) and cells were harvested after overnight incubation at 37°C. DNA was extracted using the Wizard Genomic DNA Purification Kit (Promega, USA) according to the manufacturer’s instructions. Purified genomic DNA was sequenced at SeqCoast Genomics (Newington, New Hampshire, USA) on the Illumina NextSeq2000 platform.

**Bioinformatic analyses**

Read demultiplexing, read trimming, and run analytics were performed using DRAGEN v3.10.11. Reads were also trimmed with Trimmomatic (v0.36 with default parameters) and assembled with SPAdes (v.3.15.3 with default parameters) (1, 2). The size, number of contigs, and GC content values for each genome are displayed in Supplementary Table 1. Assembled genomes were used to create an average nucleotide identity (ANI) matrix using FastANI and ANIclustermap (available at https://github.com/moshi4/ANIclustermap) (3). The phylogenetic tree based on the ANI result was edited in iTol (4). Genome sequences for Smc strains PEG-305 (GCA_009676545.1), D457 (GCA_000284595.1), K279a (GCA_000072485.1), OUC_Est10 (GCA_002138415.1), ISMMS3 (GCA_001274595.1), SmCVFa1 (GCF_001676435.1), and G51 (GCF_002799165.1) were obtained from the NCBI GenBank database and included in the ANI analysis as references for the indicated phylogenetic lineages (5). Genome sequences for strains from this study are available under the NCBI BioProject PRJNA1052082.

**Generation of the tetracycline-resistant *P. aeruginosa* strain**

A tetracycline-resistant derivative of *P. aeruginosa* PAO1 strain (PAO1 *att*Tn7::*tetR*, which is intrinsically resistant to chloramphenicol) was generated by electroporating a pUC18T-miniTn7T-Tet plasmid and a pTNS3 helper plasmid to deliver a *tetR* gene into the genome of *P. aeruginosa* PAO1. Tetracycline-resistant *P. aeruginosa* PAO1 colonies were identified by selection on LB agar plates supplemented with tetracycline (100 µg/mL). The strain was confirmed by PCR and Sanger Sequencing.

**Co-culture assays**

Overnight cultures were made in liquid LB medium from single colonies of each indicated *S. maltophilia, E. coli* DH5α*, P. aeruginosa* PAO1 *att*Tn7::*tetR,* or *S. aureus* JE2 strains and incubated at 37°C. Overnight cultures were back-diluted 1:50 in fresh liquid LB medium and incubated at 37°C for 4 hours with shaking. The optical density of all strains was set to an OD_600_ = 1 and a 1x volume of competitors (*E. coli*, *P. aeruginosa*, or *S. aureus*) was mixed with a 10x volume of the indicated Smc strains or with LB (for monocultures). 5 µL were spotted on dry LB agar plates and incubated at 37°C for 22 hours. Co-culture spots were excised, vortexed in 1 mL of liquid LB medium, serially diluted, and plated on M63 minimal medium agar (to select for *E. coli*), LB agar medium supplemented with tetracycline (20 µg/mL) or chloramphenicol (20 µg/mL) (to select for *P. aeruginosa*), or Trypticase Soy Agar with 7.5% NaCl (to select for *S. aureus*). M63 minimal medium agar plates were made by adding 15.6 g of M63 Medium Broth (AMRESCO, VWR Life Science), 15 g of agar, 1 mL of a 1M MgSO_4_ solution, 10 mL of a 20% glucose solution, and 500 µL of a 1 mg/mL thiamine solution to 1L of deionized water.

Cross co-cultivation assays to determine the potential of *P. aeruginosa*, *S. aureus*, or *E. coli* to inhibit Smc CF isolates were performed as described before with some modifications (6–8). First, *P. aeruginosa* PAO1, *S. aureus* JE2, or *E. coli* DH5α cells were set to an OD_600_ = 0.1 and spread (from left to right) on LB agar medium. Second, the indicated Smc strains were set to an OD_600_ = 0.1 and spread in a line perpendicular to *P. aeruginosa*, *S. aureus,* or *E. coli*. Plates were incubated at 37°C overnight. Growth inhibition for Smc was visually examined at the co-culture interface between the two species. Red arrows indicate observed zones of clearing indicative of inhibitory effects.

**References**

1. Bolger AM, Lohse M, Usadel B. 2014. Trimmomatic: a flexible trimmer for Illumina sequence data. *Bioinformatics* 30:2114–2120.

2. Bankevich A, Nurk S, Antipov D, Gurevich AA, Dvorkin M, Kulikov AS, Lesin VM, Nikolenko SI, Pham S, Prjibelski AD, Pyshkin A V., Sirotkin A V., Vyahhi N, Tesler G, Alekseyev MA, Pevzner PA. 2012. SPAdes: a new genome assemblyalgorithm and its applications to single-cell sequencing. *J Comput Biol* 19:455–477.

3. Jain C, Rodriguez-R LM, Phillippy AM, Konstantinidis KT, Aluru S. 2018. High throughput ANI analysis of 90K prokaryotic genomes reveals clear species boundaries. *Nat Commun* 9:5114.

4. Letunic I, Bork P. 2021. Interactive Tree Of Life (iTOL) v5: an online tool for phylogenetic tree display and annotation. *Nucleic Acids Res* 49:W293–W296.

5. Sayers EW, Beck J, Brister JR, Bolton EE, Canese K, Comeau DC, Funk K, Ketter A, Kim S, Kimchi A, Kitts PA, Kuznetsov A, Lathrop S, Lu Z, McGarvey K, Madden TL, Murphy TD, O’Leary N, Phan L, Schneider VA, Thibaud-Nissen F, Trawick BW, Pruitt KD, Ostell J. 2020. Database resources of the National Center for Biotechnology Information. *Nucleic Acids Res* 48:D9–D16.

6. Limoli DH, Whitfield GB, Kitao T, Ivey ML, Davis MR, Grahl N, Hogan DA, Rahme LG, Howell PL, O’Toole GA, Goldberg JB. 2017. *Pseudomonas aeruginosa* alginate overproduction promotes coexistence with *Staphylococcus aureus* in a model of cystic fibrosis respiratory infection. *mBio* 8:1–18.

7. Kvich L, Crone S, Christensen MH, Lima R, Alhede M, Alhede M, Staerk D, Bjarnsholt T. 2022. Investigation of the mechanism and chemistry underlying *Staphylococcus aureus*’ ability to inhibit *Pseudomonas aeruginosa* growth *in vitro*. *J Bacteriol* 204.

8. Liu Y, Gloag ES, Hill PJ, Parsek MR, Wozniak DJ. 2022. Interbacterial antagonism mediated by a released polysaccharide. *J Bacteriol* 204.
