# Supplementary material for: Antibacterial potential of Stenotrophomonas maltophilia complex cystic fibrosis isolates
Source: mSphere. 2024 Jul 9;9(7):e00335-24. doi: 10.1128/msphere.00335-24 (PMC11288042; doi:10.1128/msphere.00335-24)
Supplement: Supplemental Tables — Tables S1 and S2. [file msphere.00335-24-s0003.docx]

**Supplementary Table 1.** Relevant information for *S. maltophilia* complex (Smc) strains from this study.

| Strain Name | Patient Sex | Patient Age | Country | Year | Co-infecting  isolate | | Co-culture significance | | |
| --- | --- | --- | --- | --- | --- | --- | --- | --- | --- |
|  |  |  |  |  | **SA** | **PA** | **PA** | **SA** | **EC** |
| CCV119 | Male | Adult | USA | 2018 | No | Yes | - | *** | *** |
| CCV123 | Female | Pediatric | USA | 2018 | No | Yes | - | * | *** |
| CCV124 | Male | Pediatric | USA | 2020 | Yes | No | * | *** | *** |
| CCV125 | Female | Pediatric | USA | 2018 | No | No | - | ** | *** |
| CCV127 | Female | Adult | USA | 2019 | No | Yes | - | - | ** |
| CCV128 | Female | Adult | USA | 2019 | No | No | - | *** | - |
| CCV129 | Female | Adult | USA | 2020 | No | Yes | - | *** | ** |
| CCV130 | Male | Adult | USA | 2020 | Yes | No | - | *** | - |
| CCV131 | Male | Adult | USA | 2019 | Yes | No | * | *** | *** |
| CCV135 | Male | Pediatric | USA | 2019 | Yes | No | - | *** | *** |
| CCV139 | Male | Adult | USA | 2020 | No | No | - | - | *** |
| CCV155 | Female | Adult | Australia | 2020 | No | Yes | *** | *** | *** |
| CCV156 | Female | Adult | Australia | 2021 | Yes | Yes | - | *** | *** |

PA – *P. aeruginosa,* SA – *S. aureus,* and EC – *E. coli.* A strain was considered co-infected with *P. aeruginosa* or *S. aureus* if a positive culture of the respective bacterial species was obtained from the same pwCF at time of Smc isolation. Co-culture significance values from Figure 1 are displayed.

**Supplementary Table 2.** Genome information, prevalence of *virB10* and *virD4* Type IV Secretion System (T4SS) genes*,* as well as *tssC* and *tssM* Type VI Secretion System (T6SS) genes in the CF Smc isolates from this study.

|  |  |  |  |  | T4SS genes | | T6SS genes | |
| --- | --- | --- | --- | --- | --- | --- | --- | --- |
| Isolate Name | **Lineage** | **Contigs** | **GC%** | **Genome Size** | ***virB10*** | ***virD4*** | ***tssC*** | ***tssM*** |
| CCV119 | Sgn4 | 29 | 66.9 | 4,097,788 | - | - | 94% | 90% |
| CCV123 | Sm6 | 75 | 66.7 | 4,496,589 | 100% | 99% | - | - |
| CCV124 | Sm6 | 61 | 66.5 | 4,689,419 | 97% | 99% | - | - |
| CCV125 | Sm6 | 191 | 66.3 | 4,858,730 | 82% | 100% | - | - |
| CCV127 | Sm6 | 206 | 66.2 | 5,176,730 | 82% | 100% | - | - |
| CCV128 | Sm12 | 85 | 66.6 | 4,899,477 | - | - | 95% | 92% |
| CCV129 | Sm18 | 104 | 66.3 | 4,971,049 | 94% | 96% | - | - |
| CCV130 | Sm2 | 125 | 67.1 | 4,552,121 | - | 93% | - | - |
| CCV131 | Sm6 | 110 | 66.3 | 4,780,742 | 82% | 99% | - | - |
| CCV135 | Sm8 | 66 | 66.1 | 4,909,820 | 85% | 94% | - | - |
| CCV139 | Sm6 | 91 | 66.2 | 4,907,821 | 82% | 100% | - | - |
| CCV155 | Sm4a | 127 | 66.7 | 4,676,483 | 86% | 94% | - | - |
| CCV156 | Sm6 | 56 | 66.4 | 4,730,321 | 97% | 100% | - | - |

*virB10* and *virD4* query gene sequences were obtained from strain K279a, while *tssC* and *tssM* query gene sequences were obtained from strain STEN00241. A nucleotide megablast search with default parameters was run on the indicated CF Smc genomes from this study. The percent identity for each gene is shown. Genome sequences are available under the NCBI BioProject PRJNA1052082.
